# Fall migration, oceanic movement, and site residency patterns of eastern red bats (*Lasiurus borealis*) on the mid-Atlantic Coast

**DOI:** 10.1186/s40462-023-00398-x

**Published:** 2023-06-14

**Authors:** Michael C. True, Katherine M. Gorman, Hila Taylor, Richard J. Reynolds, W. Mark Ford

**Affiliations:** 1 Western EcoSystems Technology, Inc., 2121 Midpoint Drive, Suite 201, Fort Collins, CO 80525 USA; 2 Department of Fish and Wildlife Conservation, Virginia Polytechnic Institute and State University, 149 Cheatham Hall, 310 West Campus Drive, Blacksburg, VA 24061-0321 USA; 3 Virginia Department of Wildlife Resources, 517 Lee Hwy, Verona, VA 24482 USA; 4 U.S. Geological Survey, Virginia Cooperative Fish and Wildlife Research Unit, Virginia Polytechnic Institute and State University, 106 Cheatham Hall, 310 West Campus Drive, Blacksburg, VA 24061-0321 USA

**Keywords:** Coastal, Eastern red bats, *Lasiurus borealis*, Migration, Motus, Wind energy

## Abstract

Along the mid-Atlantic coast of the United States, eastern red bats (*Lasiurus borealis*) are present during fall mating and migration, though little is currently known about most aspects of bat migration. To reveal migration patterns, and understand drivers of over-water flight, we captured and radio-tagged 115 eastern red bats using novel technology, and subsequently tracked and described their movements throughout the region. We compared over-water flight movements to randomly generated patterns using a use-availability framework, and subsequently used a generalized linear mixed effects model to assess the relationship of over-water flight to atmospheric variables. We used hidden Markov models to assess daily activity patterns and site residency. Most bats with long-distance movements traveled in a southwesterly direction, however path vectors were often oriented interior toward the continental landmass rather than along the coastline. We observed that some bats transited wide sections of the Chesapeake and Delaware bays, confirming their ability to travel across large water bodies. This over-water flight typically occurred in the early hours of the night and during favorable flying conditions. If flight over large water bodies is a proxy for over-ocean flight, then collision risk at offshore wind turbines – a major source of migratory bat fatalities – may be linked nightly to warm temperatures that occur early in the fall season. Risk, then, may be somewhat predictable and manageable with mitigation options linking wind-energy operation to weather conditions and seasonality.

**Supplementary Information:**

The online version contains supplementary material available at 10.1186/s40462-023-00398-x.

## Background

In eastern North America, eastern red bats (*Lasiurus borealis*), hoary bats (*L. cinereus*), and silver-haired bats (*Lasionycteris noctivagans*) engage in northward movements to maternity areas in the spring and southward movements to overwintering habitats in the fall [1–4]. Mortality data from the Appalachian Mountains suggests that bat collisions with wind turbines generally aligns with this timing, and fatalities of these species are elevated during spring migration (April–May) and dramatically peaks during late summer into the fall migration and mating period (late July–October; [5–8]). Along the Atlantic Coast, where expansive offshore wind-energy development is projected to be installed [5, 6], it is plausible that bats use the coastline as a linear topographic reference for navigation and that favorable atmospheric conditions along the coast encourage coastal migration [7–9], and that the areas may also serve as mating grounds coinciding with fall migration for some species. Cryan [10] noted that easily-identifiable landmarks (such as the shape of the coastline) are used as breeding areas, heightening the concern about coastal development as a threat to the long-term population viability of the bat species in question [11–13].

Eastern red bats appear to congregate in high numbers on the mid-Atlantic and Northeastern coasts during the fall and winter using the northeast to southwest oriented Atlantic coastline as a migratory pathway [4, 14–18] for overwintering in coastal Virginia [17, 19] and (with increasing rarity) at the higher latitudes of Maryland [20], New Jersey [4], and Long Island, New York [21]. Nevertheless, tree bat migration ecology is poorly understood and therefore uncovering basic migratory behaviors such as the timing of migration events, orientation of travel, and the influence of atmospheric conditions on over-water flight would benefit the migration ecology knowledge base, but is also needed to anticipate the possible impact of collision risk with offshore wind turbines.

Historically, most information about seasonal movements of bats has been based on banding studies or accumulations of anecdotal observations [18, 22, 23]. More recently, migration in bats has been studied in three major ways: inferred seasonal movement of individuals using stable isotope analysis [24–26], the active tracking of individuals along migration routes via aircraft, drones, or ground-based vehicles using very high frequency (VHF) radio transmitters attached to bats (e.g., [27, 28]), or the passive tracking of individuals along migration routes via stationary receivers using coded radio transmitters attached to bats [27–30]. Regardless, bat migration is difficult to document because most species do not weigh enough to attach modern GPS tracking devices.

The Motus Wildlife Tracking System (hereafter, Motus; [29]) offers a novel approach to studying the migratory movements of small animals. The system uses radio-transmitters, (hereafter, nanotags or tags; Lotek Wireless,^1^www.lotek.com; Cellular Tracking Technologies, celltracktech.com) that are light enough (< 1 g) to affix to bats without impeding movement. This multi-partner, collaborative approach uses an array of ground-based VHF receiver-stations (hereafter, towers) to detect tags. As tagged animals fly within the reception distance of towers, the receivers at the towers record the tag identification (the individual), the timestamp, and the signal strength. If a tagged animal flies within range of multiple towers, some inference can be made on the coarse-scale movement paths of the individual [31]. Researchers have demonstrated the utility of using this approach for bats as direct evidence of migration, and associated patterns have been described for silver-haired bats in the Great Lakes region [29, 30, 32] as well as eastern red bats in the Northeast [27]. There is little information available on the general migratory and residency (presumably, stopover) behaviors of tree bats in the mid-Atlantic region during fall migration.

Eastern red bats, hoary bats, and silver-haired bats have been informally observed engaging in over-ocean flight during fall migration [5, 16, 33–35] and therefore presumably at risk of offshore wind turbine strikes. The mid-Atlantic is unique in geography in that it contains two large, coastal water bodies, the Chesapeake and Delaware bays. These water bodies are wide (> 50 km) at many points and therefore the drivers associated with transiting across (hereafter, over-water flight) may serve as a viable proxy for understanding drivers of over-ocean movement. Moreover, the width of these bays is comparable in distance to the distance from shoreline that many offshore wind projects in the United States are planned [6]. Motus provides the ability to infer the timing of bats engaging in over-water flight in cases when a tagged individual is detected on one shoreline and then subsequently detected on the opposite shoreline. With this information, the atmospheric conditions and within-night timing can be linked to this behavior. For instance, if temperatures decrease, or storm conditions persist, bats may use intermittent torpor during the night to reduce energy expenditure during the migratory season [36].

In recent years, there has been some investigation into the atmospheric factors that are associated with over-ocean flight to optimize conservation strategies in both bats and birds [9, 37] such as the passage of weather fronts [35], warm temperatures, low or profitable (i.e., in the direction of travel) wind speeds, and minimal precipitation [2, 16, 38, 39]. Importantly, understanding the conditions that are optimal for over-ocean flight could facilitate the creation of collision mitigation strategies such as seasonal- or condition-specific turbine curtailment protocols [27, 40–42].

During the late summers and falls of 2019 and 2021, we affixed Motus nanotags to 120 tree bats along the mid-Atlantic Coastal Plain with efforts concentrated in southern New Jersey, Delaware, and eastern Virginia. We restricted all formal analyses to the large sample of eastern red bats we gathered, but qualitatively describe movements of other migratory bat species (see Additional file 1). In our study we sought to (1) categorize and summarize the movement behaviors of eastern red bats, (2) assess the influence of atmospheric conditions relevant to over-water flight behaviors of eastern red bats, and (3) describe the general activity patterns of resident eastern red bats throughout the fall season. We hypothesized that eastern red bats migrating along the mid-Atlantic coast would travel in a southwesterly direction for fall migration. Further, we hypothesized that migratory flight might be less common and site residency times would be generally longer than observations from more northern latitudes in eastern North America due to the more favorable winter weather conditions at lower latitudes. We predicted that any movements across large bodies of water would coincide with favorable atmospheric conditions that would allow bats to minimize energy costs going into the colder months when insect prey is less active or available, whereas unfavorable conditions would be associated with prolonged periods of local residency and rest.

## Methods

### Study area

We conducted this study in the mid-Atlantic Coastal Plain regions of southern New Jersey and the Delmarva Peninsula of Delaware, Maryland and Virginia, USA in the late summer to fall periods (August–October) in 2019 and 2021. Southern New Jersey is comprised of two primary ecoregions: along the Atlantic Ocean and the Chesapeake and Delaware bay shorelines, the landscape is intertidal salt marsh and understory shrub-scrub habitat above the tidal zone [43]. Agriculture is the dominant land use on upland sites west of the coast, but mixed pine (*Pinus* spp.)-hardwood, swamp forests, and managed pine plantations are common throughout [43]. To the south, in Delaware, eastern Maryland, and the Eastern Shore of Virginia, the Delmarva Peninsula is a region bracketed by the Delaware and Chesapeake bays. Climatically, the entire study area is characterized by hot summers, and cool winters. The region has a maritime influence due to proximity to the Atlantic Ocean, meaning the annual temperature minima and maxima are less extreme relative to more inland, continental portions of the region [44]. Mean winter temperatures in the coldest month of January are above freezing at 0–3 °C for southern New Jersey and the Maryland and Delaware portions of the Delmarva, and comparatively warmer at 3–6 °C for the Eastern Shore of Virginia and the Virginia coast [45]. These same temperature ranges are experienced by eastern red bats where they have been confirmed winter residents at inland, continental sites [46].

### Motus towers, mist-netting, nano-tagging

Although there is some variation, most Motus towers are tall structural or tripod systems with two to four antennas at the top and a receiver and other electrical parts at the base [26]. Antennas are typically directional five-element, nine-element, or omni-directional and are tuned to detect VHF emissions from nanotags at 166.38 MHz [28]. After the lightweight (< 1 g) nanotags are activated and attached to animals, the tags emit this frequency and are detected by Motus towers within range [28]. Nanotags are uniquely coded transmitters that emit VHF pulses on five-, seven-, or ten-second pulse intervals (the time between pulse emissions). Calibration studies suggest an average detection range of up to 12 km for the nine-element Yagi antennas that are mounted on most towers with a clear line of sight [47, 48]. At the time of this study, Motus towers were distributed throughout North America and the array was particularly dense in sections of the mid-Atlantic (motus.org).

We captured bats in the late summer to fall periods (August–October) of 2019 and 2021 on a nightly basis starting just before sunset until three to five hours after sunset as weather permitted. We used mist-nets (Avinet, Dryden, NY, USA) of four-, six-, nine-, and 12-m widths in double- and triple-high net set configurations (i.e., heights of around ten and 15 m respectively) over trails and roads. In 2019, we spent ten nights netting in southern New Jersey (14–30 August), ten nights on the Eastern Shore of Virginia (10–23 September), and four nights in coastal Delaware (10–15 October). In 2021, we spent five nights in southern New Jersey (2–5 August and 3 September) and ten nights on the Eastern Shore of Virginia (16–31 August and 1–3 September). We netted for bats as close as possible to active Motus towers, typically < 12 km away, to maximize detection probability of bats post-release and measure the activity patterns of site residents.

During active netting nights, we identified bats to species, recorded sex (female or male), and used degree of epiphyseal fusion of the metacarpal-phalangeal joint to assign an age (adult or juvenile). We recorded other standard morphological measurements such as weight (g) and length of forearm (mm; [49]). We attached nanotags (NTQB2-1 or 2-2, Lotek Wireless, Newmarket, ON, CAN; approximate battery life of 15–30 days depending on pulse rate) using surgical cement (Perma-type surgical cement; Perma-Type, Plainville, CT, USA; approximate application retention time of 15–30 days) between the scapulae directly to the skin by parting any obstructing fur down the middle. Weight of the attached transmitter was < 5% of the individual’s body mass as recommended by Aldridge and Brigham [50]. All tagged bats were released unharmed. Our netting activities occurred under the approval of the Virginia Polytechnic Institute and State University Institutional Animal Care and Use (IACUC) protocol #19–227 and all applicable State and Federal scientific collecting permits.

### Analysis

Once all deployed nanotags were assumed to be depleted of battery and users of the Motus network had uploaded data to the database, we acquired all Motus tower detection data of deployed nanotags by using the R package *motus* [51, 52] following the data acquisition and cleaning methods of Crewe et al. [53]. Our data structure was comprised of individual timestamped detections of tags accompanied by the signal strength, the antenna, the tower location, and the tower name. These observations were accompanied by the signal strength, the antenna identifier from the tower that detected the tag, the antenna bearing, the tower location, and the tower name. We filtered out potential false-positive detections by reducing the dataset to retain detection run lengths (i.e., the number of sequential tag detections at a tower) that were > 3. We considered detections from stations > 1,000 km away from either the point of release or subsequent Motus detections as false positives, and in doing so only used detections within the general mid-Atlantic region for inference on movements. We visually inspected all paths for plausibility and filtered out detections whereby signals were picked up in improbable locations far outside the mid-Atlantic region (i.e., California). We ensured that nanotags did not simply drop off bats near towers by manually inspecting signal strength versus time and excluded the ends of deployment sections where the signal strength remained steady through time (≥ 7 days).

### Movement patterns

We examined the broad overall patterns of movement throughout the region by reporting the number of bats captured by species, sex, and age, then described any temporal trends within. We determined the proportion of bats that were detected at a Motus tower post-release. Of those bats that were detected, we noted the proportion of bats that displayed any evidence of short- or long-distance migration as proposed by Fleming [22]. We defined evidence of migration as detection at one Motus tower followed by detection at a different Motus tower > 50 km away without the return to the original or nearby detection location [22]. For those bats that migrated, we described the general direction of movement by determining the bearing of travel (from deployment location to location of the last tower detection). We calculated the range and mean bearing of travel of all bat movements collectively and displayed individual bearings graphically. We then attempted to look for evidence of the coastline as a migratory pathway by comparing the proportion of migration paths along the coastline to migration toward the interior landscape. The determination of coastline versus interior orientation of travel was defined qualitatively by visualizing tracks and determining if the ocean was immediately present to the east of the Motus stations along the track or not.

We calculated the proportion of bats that demonstrated site residency, migrated (> 50 km), or both. Site residency was defined as detections from Motus towers that were < 12 km away from the release point which contained subsequent daily detections as the bat remained in a local site > 1 night. We defined minimum site residency time as the total days between the tagging date and the last date of detection at that tower. Minimum residency times were summarized by examining the mean and 95% quantiles of the duration of individual bats’ known residency. We then described the timing of individuals engaging in migratory movement or site residency in relation to seasonal timing and demographics by visualizing density plots with respect to timing. We noted any novel, unique, or otherwise unexpected movement patterns observed in the movement paths or site residency behaviors based on our knowledge of the species’ ecology. We calculated all of the above for eastern red bats, however due to sample size constraints, we only descriptively noted the migration paths or site residency times for other tree bat species (see Additional file 1).

### Over-water behavior

We defined evidence of over-water behavior occurred as detections on two or more Motus towers that were separated in space by either the Chesapeake or Delaware bays, and within the same night. In these cases, we were reasonably certain that a bat flew above (i.e., did not transit around) either the Delaware or Chesapeake Bay. We calculated the mid-point time between across-water departures and arrivals as instances of over-water flights to the date and nearest whole hour. We considered these instances as used, or positive, points in a resource selection (use-availability) framework [54]. We created a random selection of points (10 × the number of used points) that were available to individual bats to be used as background available points. These points were restricted to instances of time between release and 40 days post-release to be reasonably certain that these instances were truly available.

Atmospheric conditions or the passage of weather fronts can influence the costs and/or benefits for birds and bats to engage in long-distance travel during migration [9, 32, 35] or, in this case, across large bodies of water. Therefore, for both used and available points, we considered the number of hours since sunset as a potentially informative variable [21]. In addition, we calculated the local atmospheric conditions which were a mix of instantaneous conditions (e.g., the current wind speed and temperature) or changes in conditions (i.e., an increase in pressure indicating the passage of a weather front). We selected instantaneous (hourly means) conditions of wind speed (m/s), temperature (°C), visibility (0–16 km), precipitation (accumulated cm), the longitudinal component of wind speed and direction (m/s), the latitudinal component of wind speed and direction (m/s), as well as one-hour and 24-h changes in these components (Table 1). As a surrogate variable to indicate the passage of weather fronts [55], we created a set of delta (∆) variables. These variables were calculated as the change from one- or 24-h increments including the one-hour change in wind speed (m/s), the one-hour change in temperature (°C), and the 24-h change in pressure (∆kPa; Table 1). For all measurements of atmospheric condition variables, we used the nearest available weather station available on Visual Crossing (visualcrossing.com/weather-data; Visual Crossing, Hamburg, Germany; accessed 1 October 2021) which ranged from five to 20 km from Motus towers involved in this portion of the analysis.

**Table 1:** The variables, explanations, and expected associations used in modeling the relative probability of over-water flight by eastern red bats (*Lasiurus borealis*) in the mid-Atlantic using data collected mid-Atlantic Coastal Plain in the fall of 2019 and 2021

Variable	Explanation	Expected association
Wind speed	Hourly wind speed (m/s)	–
Temperature	Hourly dry bulb temperature (°C)	+
Precipitation	Hourly precipitation accumulated (cm)	–
Visibility	Hourly visible distance (0–16 km)	+
Pressure	Hourly Barometric pressure (kPa)	
Longitudinal component of wind speed and direction	The longitudinal (*x*) component of the hourly wind speed (m/s) and direction (0–360°) vector. Positive values are easterly winds, negative values are westerly winds	–
Latitudinal component of wind speed and direction	The latitudinal (*y*) component of the hourly wind speed (m/s) and direction (0–360°) vector. Positive values are northerly winds, negative values are southerly winds	–
∆ Wind speed 1-h	The change in wind speed (m/s) from the previous hour to the current hour	–
∆ Temperature 1-h	The change in temperature (°C) from the previous hour to the current hour	+
∆ Pressure 24-h	The change in nightly pressure average (∆ kPa) from the previous night to the current night	+

We created a generalized linear mixed model with a logit link function [56, 57] in the R package *lme4* [58] using the binary response of used (positive) instances of over-water flight and available instances of suspected non-flight (negative). We treated hours since sunset and the atmospheric variables as fixed effects and a unique nanotag ID as a random effect. No variable reductions were necessary due to multicollinearity. We performed a dredge using R package *MuMIn* [59], which created a large set of independent models composed of all possible additive combinations of variables, ranked each model by AICc [60], and retained all models within < 2 ∆AICc units as competing models [61]. Within this set of models, we selected the top model based on our understanding of migratory bat ecology [60]. To visualize the relative probability of over-water flight by eastern red bats we calculated marginal effects for variables included in the top model. Marginal effects are predictions (and 95% predictive intervals) of the relative probability given a range of values for an explanatory variable while keeping all other variables at their means. We then plotted these effects and visualized them graphically.

### Site residency daily activity patterns

We were able to collect consistent timestamped information on signal strengths when the bat engaged in site residency. Signal strength readings increase when a tagged bat is closer to a tower or when the distal end of the tag is oriented toward the tower. When a resident tagged bat was actively flying, the variability in the signal strength was expected to be higher because the attached transmitter was changing angles, heights, and distances in relation to the receiving tower and antennas [62].

To provide insights on the nightly and seasonal activity patterns of bats that retain site residency in the fall, we reduced the entire dataset to contain only bats that retained site residency for > 20 days, had consistent readings on a single or few towers in close proximity, and with not more than two sequential days without detections. We scaled signal strength readings by dividing tower- and antenna-specific data by their standard deviations for consistent scales across towers. We then binned signal strength readings of individuals by hour and calculated the hour-by-hour standard deviations of scaled signal strengths across individual antennas. If the bat was recorded on multiple antenna units from a tower, we averaged the signal strength standard deviations across all antennas.

To estimate the underlying state (resting or active) during the time series of detections for each bat, we used hidden Markov models (HMMs; 63). HMMs decouple the relationship between an underlying ecological process (e.g., a time series of discrete states such as rest or active) and the potentially noisy observations that result due to a time series of continuous observations. Because the time series of ecological states are not directly observable, the states are inferred as hidden or latent based on the observations. We used the R package *depmixS4* to create two-state HMMs [63]. The HMMs in this R package are refined such that the transition matrix can be regressed (generalized logistic with a logit link function) against time-varying covariates. In this case, the probability of transitioning between active and resting states can vary with the diurnal cycle of arousal and resting/roosting [64]. We modelled each bat individually because each bat and tower combination is unique, and the model cannot incorporate random effects.

We fit each model using signal strength standard deviations as the response and used transition matrix covariates of cyclical transformed hours since sunset (0–23), that involved both sine and cosine transformations which varied sinusoidally from -1 to 1 on a 24-h period. We used this variable to increase model stability signifying the diurnal and nocturnal patterns of activity and rest. Once the models were fit, we predicted the underlying states for each hour using the function *posterior* in *depmixS4* using the Viterbi algorithm to predict the likely state given the data [65]. We then filtered the data to contain night hours only and plotted each hourly state prediction against variables of date, hours since sunset, wind speed, and temperature, to visualize the conditions in which bats are most likely active or resting during night hours.

## Results

### Overall patterns

**Fig. 1:**
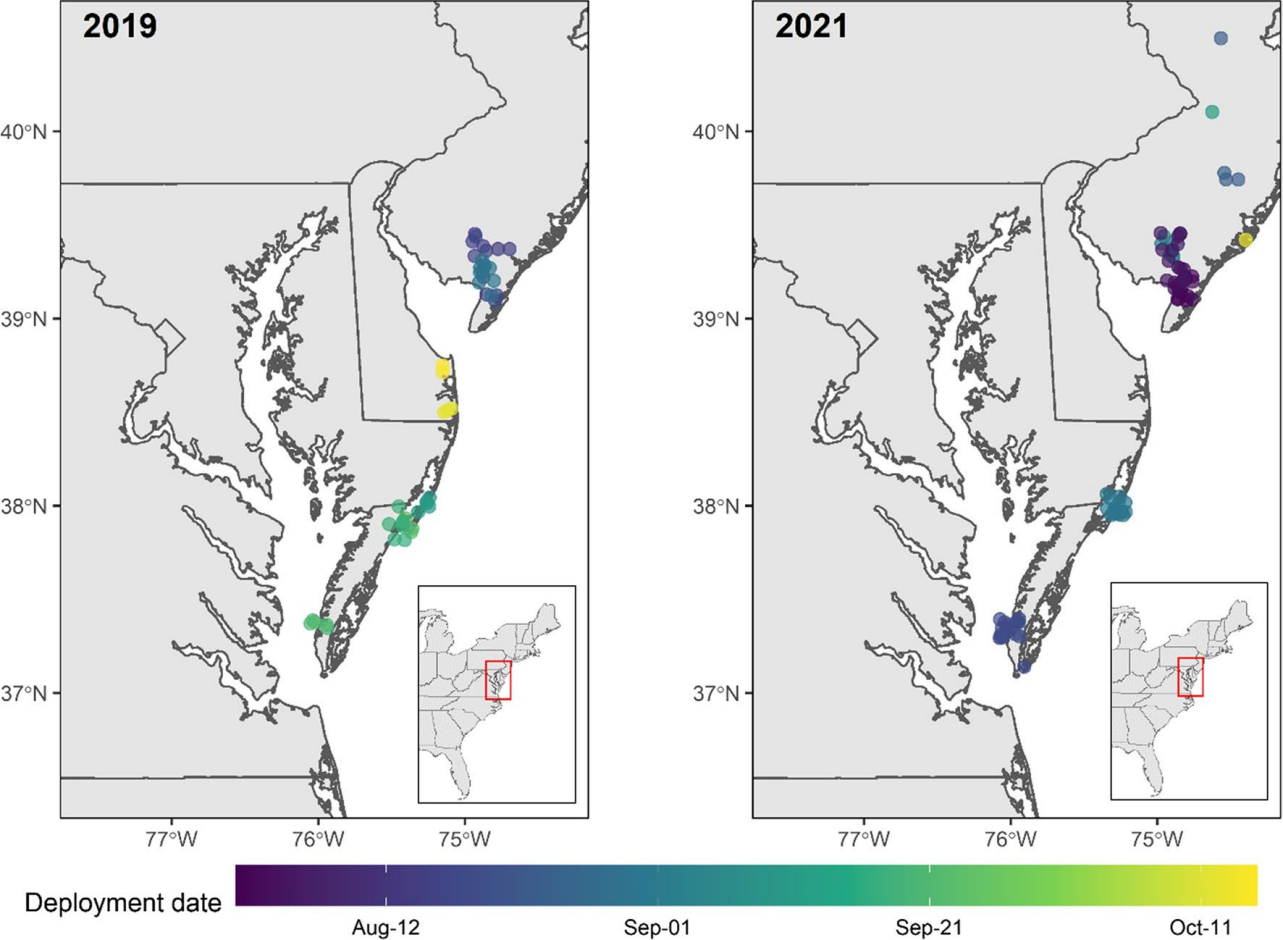
Locations and dates of active bat netting and deployment of nanotags on eastern red bats (*Lasiurus borealis*), hoary bats (*L. cinereus*), and silver-haired bats (*Lasionycteris noctivagans*) within mid-Atlantic Coastal Plain region of the United States in the late summer to early fall period of 1 August–15 October in 2019 (left) and 2021 (right). Generally, over the two years, netting efforts began in southern New Jersey and continued down the coast to finish on the Eastern Shore of Virginia. Delaware was the last locality sampled in 2019

In the late summer to fall periods (August–October) of 2019 and 2021, we caught and tagged 120 bats of our three focal species throughout the coastal mid-Atlantic study area (Fig. 1 and Table 2). In 2019, we tagged 22 eastern red bats in southern New Jersey, 19 eastern red bats and one Seminole bat (*L. seminolus*) on the Eastern Shore of Virginia, and four eastern red bats and one Seminole bat in Delaware (Table 2). In 2021, we tagged 40 eastern red bats and two silver-haired bats in southern New Jersey, and 30 eastern red bats and one Seminole bat on the Eastern Shore of Virginia (Table 2). Over both years, we tagged 31 (26%) bats from 1–14 August, 55 (46%) bats from 15–31 August, 13 (10%) bats from 1–14 September, 15 (12.5%) bats from 15–30 September, and six (5%) bats in October.

**Table 2:** The locations of eastern red bats (*Lasiurus borealis*), hoary bats (*L. cinereus*), and silver-haired bats (*Lasionycteris noctivagans*) captured and tagged in the mid-Atlantic Coastal Plain region of the United States (Fig. 4) in the fall periods (August–October) of 2019 and 2021

Year	Location	Number of tagged bats		
		Eastern red	Silver-haired	Seminole
	Southern New Jersey	22	0	0
2019	Coastal Delaware	4	0	1
	Eastern Shore of Virginia	19	0	1
2019 Total	All	48	0	2
2021	Southern New Jersey	40	2	0
	Eastern Shore of Virginia	30	0	1
2021 Total	All	70	2	1
Grand total	All	116	2	3

In total we captured 116 eastern red bats, two silver-haired bats, and three Seminole bats (*L. seminolus*)

### Eastern red bats

**Fig. 2:**
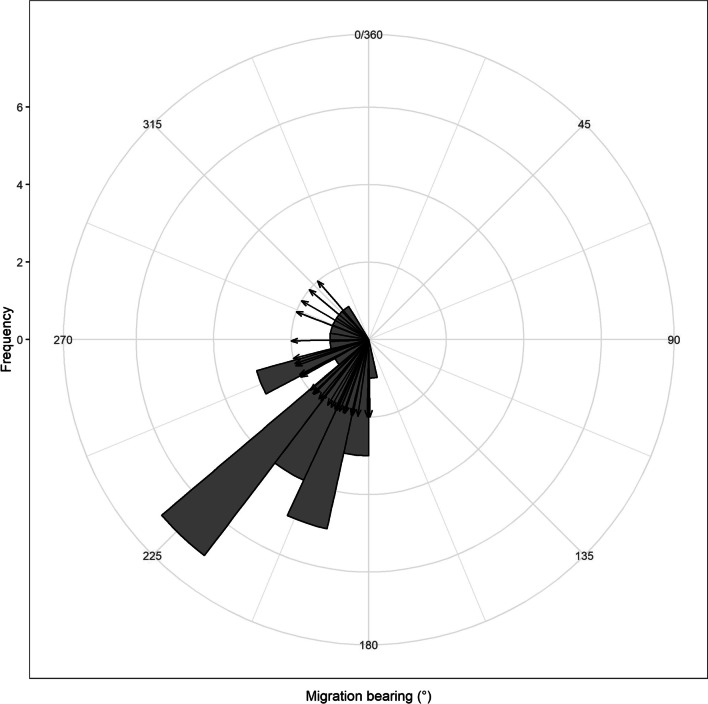
The bearings of eastern red bats (*Lasiurus borealis*) from their point of origin to their final detected destination based on detections from towers in the Motus Wildlife Tracking System mid-Atlantic Coastal Plain of the United States during fall of 2019 and 2021. This is displayed as an angular (0–360°) histogram which shows the frequency of bearings in histogram bins (gray boxes). The raw data (arrows) are displayed showing the flight bearings

One hundred fifteen tagged eastern red bats accounted for approximately 95% of the total migratory tree bat captures. The sex ratio (females to males) increased temporally through the tagging periods but, overall, we captured more males (*n* = 75; 65%) than females (*n* = 40; 35%). Most bats were adults (*n* = 74; 64%) as opposed to juveniles (*n* = 39; 34%) or unknown (*n* = 2; 2%). Of the adults, 30 were female (41%) and 44 were male (59%). Of the juveniles, 10 were female (26%) and 29 were male (74%). The two bats with unknown ages were male. Post-release, 95 bats (83%) were detected by at least one Motus tower in the region. Detection probability was related to proximity to a Motus tower as 83 bats (93%) were detected when released near a tower. In contrast, only 12 bats (46%) were detected when released > 12 km from the nearest Motus tower. Of the 95 eastern red bats detected, 29 individuals (31%) displayed migration behavior. The direction of migration was southwesterly as the mean bearing of travel was 230° (min = 179, max = 319; Fig. 2). Bearings were concentrated in a southwesterly direction, but some directly south and northwest bearings of travel existed (Fig. 2). Bats displayed both inland and coastal orienting when moving through the region. Most migrating bats displayed the former approach such that 17 bats (58%) chose a route directed inland whereas 12 bats (42%) took a coastal route. Three bats tagged in southern New Jersey appeared to have circumvented the Delaware Bay by travelling north along the coastline instead of crossing the bay and travelling south (one adult female and two juvenile males). The last detection locations of individual bats that showed evidence of migration ranged from coastal North Carolina (*n* = 2; 6%), the Eastern Shore of Virginia (*n* = 6; 19%), west of the Chesapeake Bay in Virginia or Maryland (*n* = 14; 45%), on the Delmarva Peninsula in Maryland or Delaware (*n* = 7; 23%), and New Jersey (*n* = 2; 6%).

For bats that were tagged near Motus towers, 84 individuals (89%) were residents post-release. Mean minimum site residency time was 15 days (0.05 and 0.95 quantiles were two and 32 days, respectively). Site residency time as a point estimate was slightly lower for bats tagged in September (average of 11.3 days) versus those tagged in August (average of 15.5 days). There were no obvious trends in migratory status or minimum residency time in relation to sex or age in our sample of tagged bats as the proportions were relatively even for each documented behavior. We observed a novel behavior in some bats (*n* = 7; 6%) whereby a small proportion engaged in long distance travel (> 50 km), were documented on multiple non-local Motus towers and, in some instances, later returned near the original tagging site.

### Over-water behavior

**Fig. 3:**
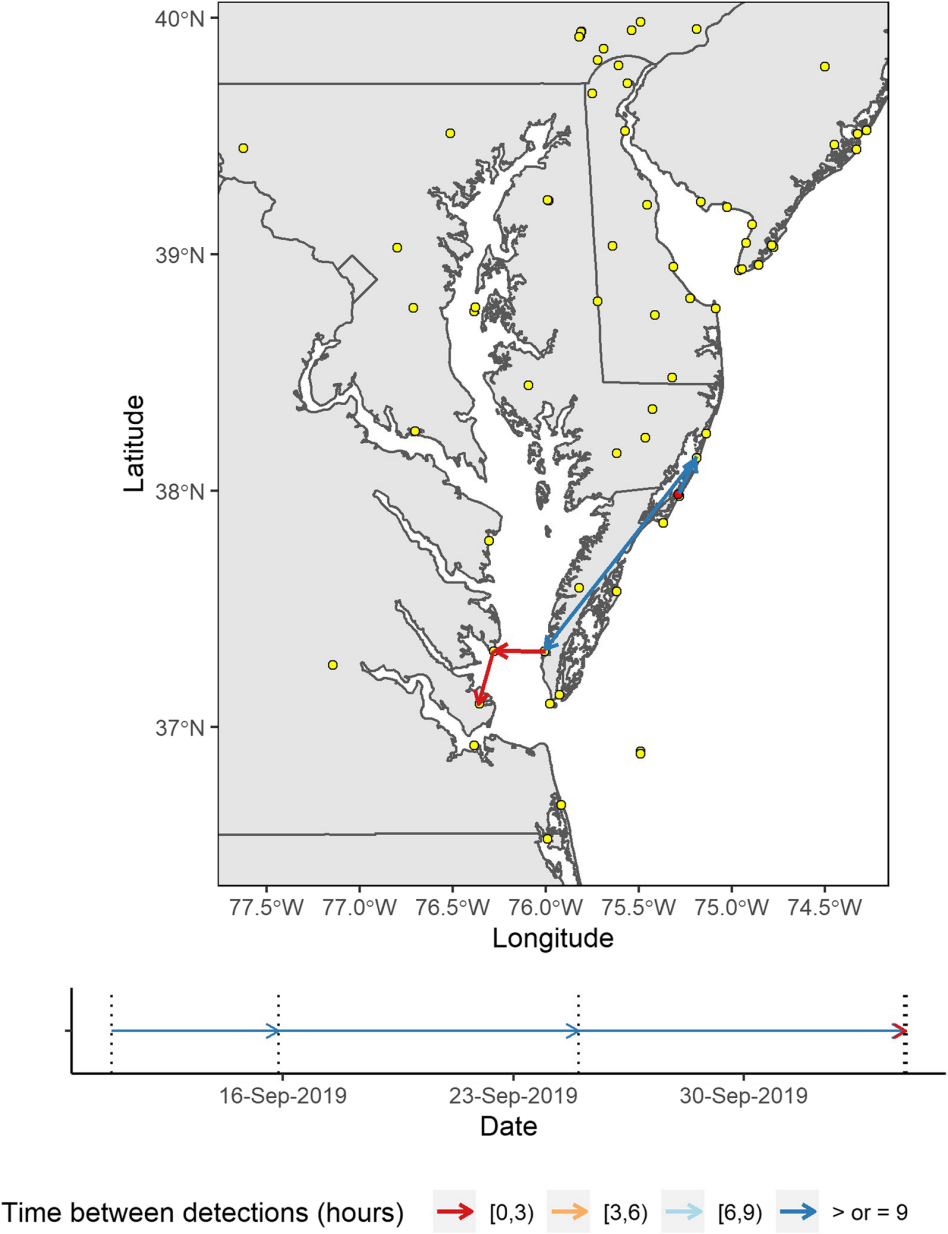
The migratory route of a tagged adult female eastern red bat (*Lasiurus borealis*) displaying an over-water flight behavior. This map connects the coarse locations of the individual by connecting detections at Motus towers by line-segmented arrows (arrow indicates direction of travel, colors represent time lag, in hours, between detections, and yellow points are Motus towers). This bat was tagged on 10 September 2019 on the Eastern Shore of Virginia and moved across the Chesapeake Bay in less than three hours on 4 October 2019

**Fig. 4:**
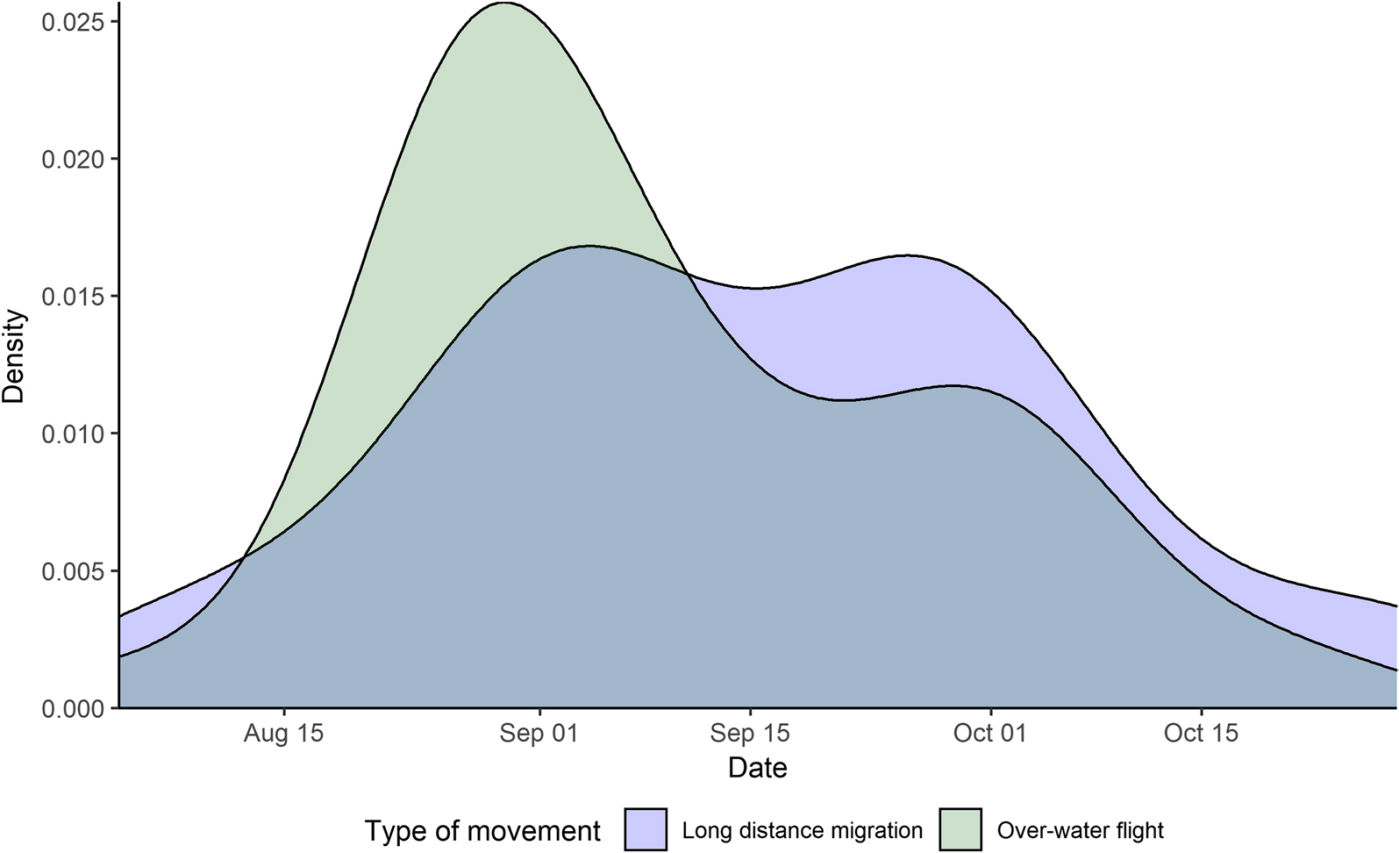
The density of long-distance migration events (blue curve) and over-water flights (green curve) and as they relate to time of the year from Motus telemetry data on eastern red bats (*Lasiurus borealis*) in the mid-Atlantic Coastal Plain in falls of 2019 and 2021. Over-water forays distinctly peaked in late August to early September while long distance migration events were sustained from late August to early October. Note that tagging events were not evenly distributed throughout this period

We detected numerous instances of eastern red bats transiting across the Chesapeake and Delaware bays (single night flights as seen in Fig. 3). Of the 95 bats that were detected at Motus towers, 31 bats (32%) displayed over-water behavior. These movements were not necessarily migratory as some bats crossed the body of water, then returned to the original detection locality either the same night or some night after. This over-water flight, as opposed to explicit migratory behavior, appeared to be loosely seasonally dependent as over-water behaviors peaked in the late August to early September whereas explicit migration events occurred relatively evenly throughout August to October (Fig. 4).

**Fig. 5:**
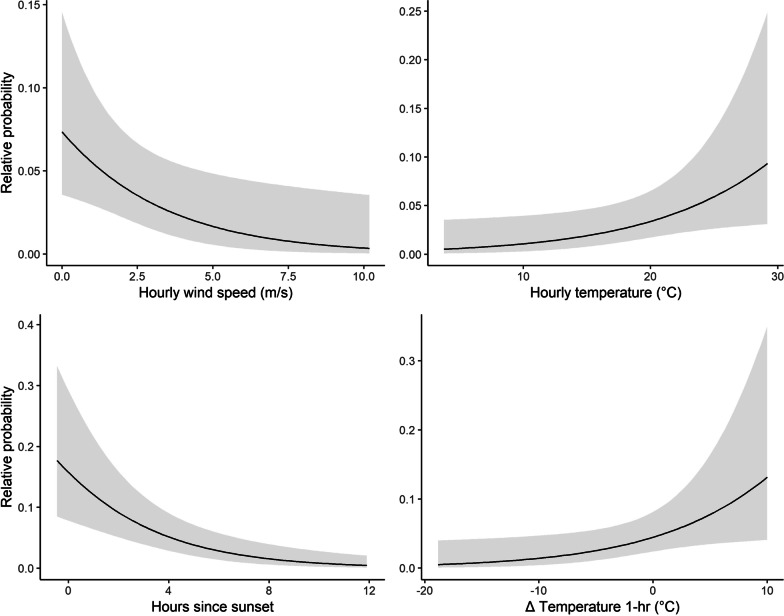
Selected marginal effects of the use-availability model of over-water flight given atmospheric conditions to visualize the effect of significant variables. Marginal effects are predictions of the relative probability of over-water flight of bats based on a single variable [each above] while holding all other variables at their means. The prediction estimate is displayed (black line) along with the 95% prediction interval (gray band)

We detected 39 instances of over-water flight for used (positive) instances in the use-availability logistic models. We used 390 available (negative) instances for potential non-use. We compared 1,024 individual models assembled with the dredge function and found that ten models were within two ∆AICc points. Our top model included effects of wind speed and hours since sunset which were negatively associated with use and included effects of longitudinal (*x*) component of wind speed and direction, latitudinal (*y*) component of wind speed and direction, and one-hour change in temperature which were positively associated with use (Table 3). Over-water travel was statistically positively related to temperature, one-hour change in temperature, and the latitudinal (*y*) component of wind speed and direction. Over-water travel was statistically negatively related to wind speed and hours since sunset. We visualized a negative trend between over-water flight, wind speed, and hours since sunset, and found a positive association between use and both temperature and the change in temperature over a 24-h period (Fig. 5).

**Table 3:** Parameters, beta (β) estimates, standard errors (SE), *Z-*statistics, and approximate significance (*p*-values) of the top approximating model formulated from data collected on eastern red bats (*Lasiurus borealis*) exhibiting over-water flight behaviors across the Chesapeake or Delaware bays in the fall seasons of 2019 and 2021

Parameters	*β*	SE	*Z-*value	*p*-value
Intercept	− 2.92	1.36	− 2.14	0.032
Hours since sunset	− 0.31	0.08	− 3.08	< 0.001
Wind speed	− 0.31	0.14	− 2.26	0.024
Temperature	0.12	0.06	2.11	0.035
Longitudinal component of wind speed and direction	0.18	0.11	1.62	0.104
Latitudinal component of wind speed and direction	0.46	0.12	3.94	< 0.001
∆Temperature (one-hour)	0.12	0.55	2.15	0.031

### Site residency daily activity patterns

We observed four adult male eastern red bats that retained site residency for ≥ 20 days and for which there was a constant collection of timestamped signal strengths from a nearby Motus tower. Two of these bats were tagged in 2019 and two were tagged in 2021. The data on timestamped signal strengths were recorded at the Motus tower at Savage Neck Natural Area Preserve, Northampton County, Virginia beginning on 21 September 2019 (for one bat) and 18 August 2021 (for two bats) and at the Motus tower at Cape Henlopen State Park, Sussex County, Delaware beginning on 14 October 2019 (for one bat).

**Fig. 6:**
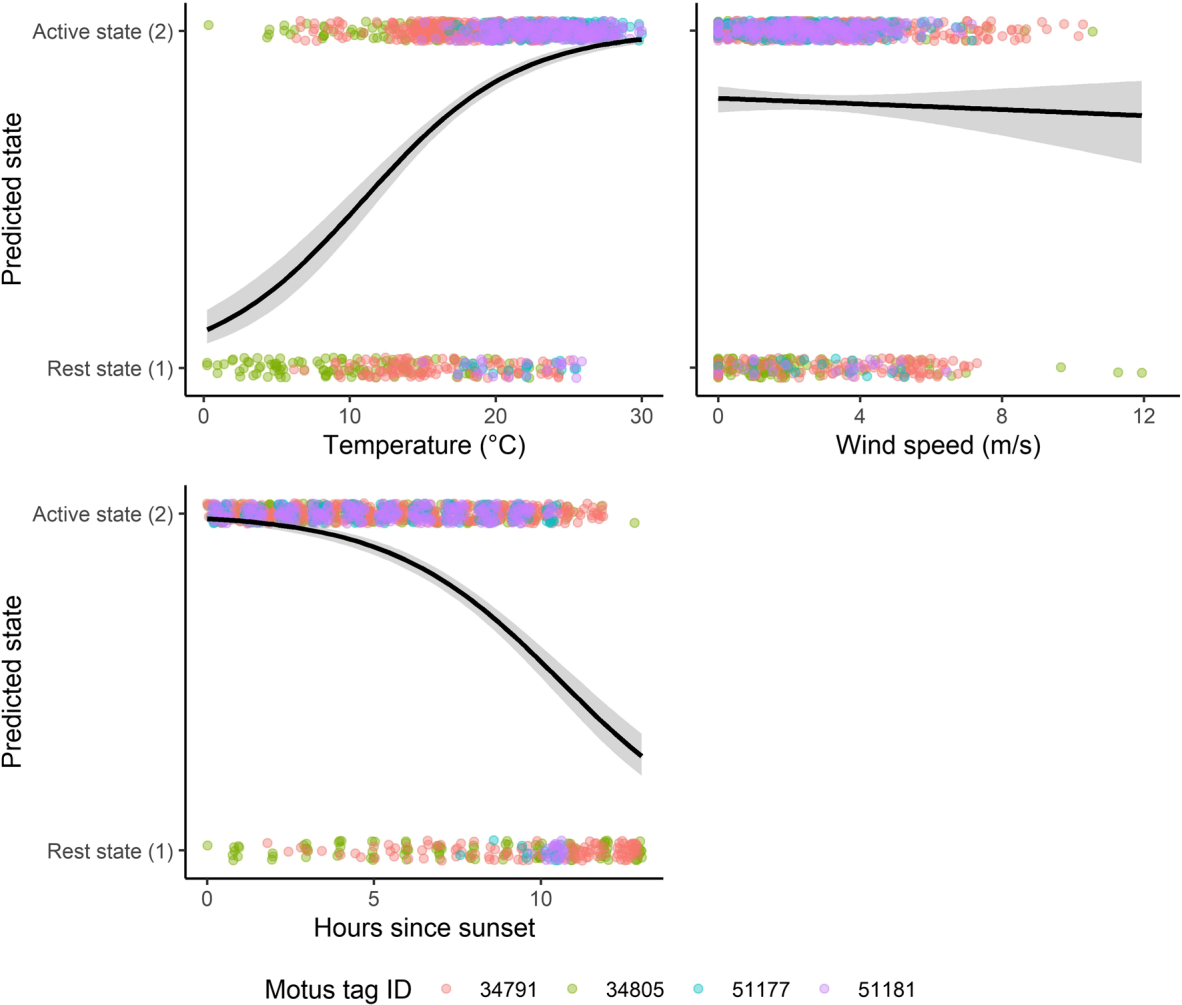
The predicted state (rest [1], active [2]) of individual bats (*n* = 4; pink, green, orange, and blue points) from the hidden Markov models (HMMs) formulated from Motus telemetry data on eastern red bats (*Lasiurus borealis*) on the Delmarva Peninsula portion of the mid-Atlantic Coastal Plain in the fall seasons of 2019 and 2021. Individual points’ states at hourly intervals during the night versus atmospheric conditions (temperature and wind speed; top row) and hours after sunset (bottom left). Points are given a small amount of random variation for aid in visualization. Logistic regression lines (black) show the general trend in the state in relation to the variable of interest

We found a general decreasing trend in the proportion of predicted hourly active versus rest states as time progressed from August to November. The bats tagged in August were active on nearly every night hour over the life of the tags. Conversely, the bats tagged in late September and October were considered to be active during a lower proportion of night hours over the life of the nanotags. We noted a short period of decreased proportion of night hours when bats were predicted to be in an active state which correlated directly with temperatures falling from approximately 15–20 °C to < 10 °C. The proportion of active to rest states increased with temperature and decreased with hours after sunset and wind speed (Fig. 6), though the effect of wind speed was weak.

## Discussion

Eastern red bats along the mid-Atlantic Coast behaved in similar but at times contrasting ways to those of a similar study on the species that occurred at higher latitudes of the Northeast [27]. For instance, both in our study and in Dowling [27], a similar proportion of tracked eastern red bats (30%) engaged in long-distance migration; however the direction of travel (bearing), the timing, and the pathway of travel (coastal or inland) were highly variable. We expected the bats tagged in our study to show a lower proportion of migration because of the more southerly latitude and known over-wintering residency within the study area. Our results confirmed that, within the study period, most of the eastern red bats we tracked were not actively moving long distances.

Nonetheless, eastern red bats showing clear evidence of migration did so with some degree of consistency. Some bats moved slightly north and west, presumably to avoid crossing large sections of either bay, however most bats in the study moved through the region in a southwesterly direction. These observations also were consistent with Dowling [27] in that bats from the Northeast also travelled in a southwesterly direction, although our study included some northbound outliers. This effect could be explained by the fact that the geographic distance between two potential barriers, the coastline, and the Appalachian Mountains, are closer in proximity in the Northeast than in the mid-Atlantic and thereby funnel bats closer to the coast. Accordingly, migrating bats may be able to disperse more widely (i.e., have a wider range of possible bearings of travel) once they reach southern New Jersey or the Delmarva Peninsula because the distance between the movement-restricting coast and mountains is larger. The lack of coastal movements in the mid-Atlantic may suggest that the Northeast coastline is used by a higher proportion of the regional population of eastern red bats than the mid-Atlantic coastline.

Despite moving in a general southwesterly direction, less than half of migrating bats that we documented explicitly used coastline as a migration route and instead oriented toward the interior of the landmass. This may challenge traditional assumptions that bats use linear features as a reference for migration (at least, the linear feature of the coastline). Recent studies have also concluded that inland bats do not necessarily use linear features such as rivers to the degree previously thought [66, 67]. This assumption remains operative [4] because of greater than expected seasonal increases in activity along the mid-Atlantic [2, 39]. Nevertheless, eastern red bat use of the shoreline in the Northeast may be higher proportionately than the mid-Atlantic because the region offers a larger and broader landscape suitable for migration, leading to a more widely distributed population in fall.

Eastern red bats in our study did have higher average minimum site residency times (mean = 15 days; SD = 9.2 days) than bats in the Northeast (6.9 days; 18). This finding is interesting in that residency times were longer than we would expect for a temporary stopover, and may support the point that, at the more southerly latitudes, either the need to migrate is simply less critical, or large-scale movements happen at later dates than when we tracked bats. Certainly, the mid-Atlantic is warmer and is more suitable for over-winter use than the Northeast [4], allowing bats to engage in lengthy site residency times during the months we tagged them (in August and September) or to travel a southwesterly distance later in the year if they did not reside locally through the winter.

We demonstrated that eastern red bats can travel over large bodies of water, suggesting some level of collision risk at offshore wind turbines is possible. Over-water flight appeared positively influenced by warm temperatures and low wind speeds, which was expected as these are two atmospheric conditions that have been shown to influence activity rates of offshore bats [16, 38, 39, 68]. In addition, the nightly timing of these events was informative as over-water flight behavior occurred typically in the early hours following sunset. This effect has been documented with bat activity onshore in that peak activity occurs directly following sunset (e.g., [21]). For bats offshore, there are more mixed results including activity occurring before sunset [16] and in the early daylight hours [69]. We did not find pressure change as proxy to the passage of a cold weather front to be an informative predictor in the model as in Cryan and Brown [35]. Alternatively, it could be interpreted that one-hour change in temperature may be indicative of the clearing of cold fronts as associated with over-water flight.

The seasonal timing of over-water events generally peaked in late August to early September, whereas directed, explicit migration events were relatively evenly distributed throughout September when individual bats were equally able to demonstrate either or both. On Virginia’s barrier islands, True et al. [39] observed a similar effect in that barrier island visitation (which implies some degree of over-water flight from the mainland across the sound) explicitly peaks in the late summer (around 19 August). Similarly, in the same area, the over-ocean observations of eastern red bats in a study by Hatch et al. [69] occurred generally in early September. This effect could simply be a product of the generally warmer period in August rather than late September. Indeed, we demonstrated that the proportion of night hours in a foraging, or active, state decreased as the season progressed to October and late fall, so it is plausible that over-water flights could peak seasonally during periods when insect prey was still abundant and able to support the caloric demands of over-water flight. In addition, energetic costs associated with over-water flight may also be minimized by the generally warm period of late August.

Although it remains unknown whether over-water movement over the Chesapeake or Delaware bays is a viable proxy for over-ocean movement, our results are consistent with other observations [2, 16, 35, 39, 68]. Combinations of low wind speeds, high temperatures, passage of storm fronts, and/or early fall seasonality are most associated with high probabilities of bats engaging in open water flight [5]. Accordingly, wind turbine collision by bats may be highest during these conditions and operational curtailment such as increasing of minimum wind speed thresholds or rotating wind turbine blades to avoid or reduce the number of bat fatalities annually could be effective for offshore facilities as demonstrated from inland facilities [27, 70–72].

Informed and focused curtailment during periods of expectations of high risk (referred to as smart curtailment) is increasingly being employed by energy producers [40, 41, 73]. For instance, at its simplest implementation, a wind operator might curtail during the fall and minimize considerable mortality risk, as that is the season associated with highest collision risk [39]. A more complicated curtailment algorithm could include temperature or other weather conditions in setting nightly or weekly curtailment. At offshore wind facilities, managers could use the conditions that we found informative to set curtailment standards at periods of expected risk in a relatively cost-efficient manner [27, 42]. Our study did not explicitly assess risk to collision strike at wind facility localities and therefore we suggest wind turbine managers first monitor for bats at facility-specific localities – it could be that relative to interior wind-energy sites, migratory tree bat passage over the open ocean at planned deployment distances is relatively rare. Nevertheless, our study provides the likely conditions in which over-water flight is possible, serving as a starting point for identifying specific periods when wind-energy managers could focus monitoring efforts as well as what to expect for the atmospheric, within-night, and within-season influences of conditions on collisions risk at offshore wind facilities in the mid-Atlantic.

## Conclusions

Our study marks the largest and first effort to date to study eastern red bat fall migration patterns along the mid-Atlantic coast with automated telemetry. Our hypotheses were generally supported in that eastern red bats moved throughout the region in a common southwesterly direction. However, although some bats showed evidence of migration, other bats displayed lengthy site residency often encompassing the entire duration of the expected tag life or time to shedding their tag. This may suggest that in the mid-Atlantic there is a delayed urgency, or no need, to migrate at least in the early to mid-fall period, although this hypothesis for the months of October and November remains untested with an appreciable sample size. We documented eastern red bats traveling across both the Chesapeake and Delaware bays and, as hypothesized, they did so relative to atmospheric conditions and within-night timing – potentially to reduce energy expenditure. Lastly, we provided evidence that bats that engage in site residency will switch to rest states (and perhaps torpor bouts) at times throughout the night during periods of low temperatures (and the progression of the fall season), similar to stop-over bouts of silver-haired bats in the Great Lakes region [30, 36]. As wind turbine development progresses offshore, a better understanding of the underlying biological drivers and patterns of movements throughout the continent for migratory bats would be contributory [74], particularly as some species may become imperiled due to wind-energy development [13, 75]. Although our study made progress documenting migratory patterns, the development and execution of similar studies in the mid-Atlantic, other regions in the United States, and abroad could help inform the conservation and management of migratory tree bat species in an era of increasing wind energy development [76–81].

## Supplementary Information

**Additional file 1. **Results for silver-haired bats and Seminole bats.

## Data Availability

Data are available from the U.S. Geological Survey https://www.sciencebase.gov/catalog/item/640766d9d34e76f5f75e385f.

## Footnotes

1 Any use of trade, firm, or product names is for descriptive purposes only and does not imply endorsement by the U.S. Government.
