# Supplementary material for: Fall migration, oceanic movement, and site residency patterns of eastern red bats (Lasiurus borealis) on the mid-Atlantic Coast
Source: Mov Ecol. 2023 Jun 14;11:35. doi: 10.1186/s40462-023-00398-x (PMC10268339; doi:10.1186/s40462-023-00398-x)
Supplement: Supplementary file 1 — Additional file 1. Results for silver-haired bats and Seminole bats. [file 40462_2023_398_MOESM1_ESM.docx]

**Supplementary Materials**

**Results for silver-haired bats and Seminole bats**

We also captured silver-haired bats (*n* = 2; adult females) and Seminole bats (*n* = 3; adult females). For these two species, we documented some degree of migratory and site residency behavior but did not conduct a formal analysis due to small sample size.

One silver-haired bat was tagged and released on 3 September 2021 but was never detected upon release in southern New Jersey at Peaslee Wildlife Management Area, Cumberland County, New Jersey. Another silver-haired bat was tagged and released on 13 October 2021, in southern New Jersey in Ducktown, Atlantic County, and moved along the Atlantic coastline, across the Delaware Bay, eventually transiting inland to Maryland by late October. We tagged two Seminole bats on 21 September 2019, and 31 August 2021, on the Eastern Shore of Virginia at Savage Neck Natural Area Preserve, Northampton County, Virginia, and Chincoteague National Wildlife Refuge, Accomack County, Virginia, respectively. However, there was no evidence of migratory behavior for these bats. They displayed site residency for eight and 17 days, respectively, before contact was lost. One additional Seminole bat was captured on 10 October 2019, in southeastern Delaware at Assawoman Wildlife Management Area, Sussex County, Delaware and migrated south along the Atlantic coastline on the evening of 28 October 2019.

Although we were not able to describe the overall patterns of migration with respect to timing, direction, and degree of over-water flight for these species due to low capture rates, the two silver-haired bats and three Seminole bats that we tagged do provide some anecdotal information.

Our success in only capturing two silver-haired bats, was unexpected because they are a species often noted offshore along the Atlantic Coast (second only to eastern red bats; [38]). The mid-Atlantic Coast is suitable for silver-haired bats during the fall (4) and they have historically been observed in the region during the fall and winter (1). However, our capture effort was focused primarily in August and September, so it is possible that silver-haired bats may have not have arrived in appreciable numbers from their northern maternity grounds when we mist-netted. Moreover, the bulk of silver-haired bat winter distribution is predicted to be more northerly (5,8) than our study area which may therefore explain the limited captures. Considering this, offshore wind collision risk, at least in the mid-Atlantic, may be lower or delayed for silver-haired bats compared to that of eastern red bats. However, we documented migration and an over-water flight behavior in one silver-haired bat in late October that had been tagged along the New Jersey Atlantic coast, so that risk cannot entirely be discounted.

Seminole bats are not common in mid-Atlantic region as their range is thought to be largely restricted to southeast Virginia (3). True et al. (81) observed that due to a changing climate and suitable day-roost habitat, the mid-Atlantic Coast may be more suitable for the species during the active season than previously thought. Perry (3) noted that Seminole bats exhibiting migratory behaviors are corroborated by the evidence of a southward transiting Seminole bat along the Atlantic Coast in late October.
